# Participatory Design and Evaluation of the “Stem Cells Australia” Website for Delivering Complex Health Knowledge: Mixed Methods Study

**DOI:** 10.2196/44733

**Published:** 2023-07-20

**Authors:** Patrick Cheong-Iao Pang, Megan Munsie, Shanton Chang, Claire Tanner, Christine Walker

**Affiliations:** 1 Faculty of Applied Sciences Macao Polytechnic University Macao Macao; 2 Melbourne Medical School The University of Melbourne Parkville Australia; 3 Stem Cell Ethics and Policy Group Murdoch Children’s Research Institute Melbourne Australia; 4 School of Computing and Information Systems The University of Melbourne Parkville Australia; 5 School of Social Sciences Monash University Melbourne Australia

**Keywords:** stem cells, health websites, health information–seeking behavior, complex health information, participatory design, public health

## Abstract

**Background:**

The internet has become a commonly used information source for people seeking to understand their health care options. However, inconsistent representation about what stem cell treatments are available and from whom, coupled with the lack of transparency about what has been shown to work or is even safe, can distract and mislead users. Given these challenges, there is a need to develop effective evidence-based tools for delivering information about health care options involving stem cells.

**Objective:**

A need to redesign an existing website in Australia was identified to provide trustworthy information about stem cell research and to counter misinformation. Using a participatory design approach to generate an understanding of what information users need for stem cell treatments, the Stem Cells Australia website serves as a case study for the development and evaluation of websites delivering complex messages about science and health.

**Methods:**

This study comprised 3 steps. First, a focus group and several one-on-one interviews with a purposive sample of users (n=12) were conducted to identify their needs and requirements. Second, we designed a new version of the website based on findings from the focus group and interviews. Finally, for evaluating the participatory design process, we collected 180 days of Google Analytics data for both the original and redesigned versions (90 days for each) and compared their differences using 2-sample *z* tests.

**Results:**

The feedback from participants was grouped into 3 themes—needed and unwanted information, how and where to obtain information, and their information preferences. These were translated into requirements for rebuilding the website. The redesigned version reached users in other continents, despite the daily numbers of users (−61.2%; *P*<.001) and sessions (−61.7%; *P*<.001) decreasing. The redesigned version also showed substantial decrease in daily bounce rate (−97.2%; *P*<.001), significant increase in the daily average of page reads per session (+110.8%; *P*<.001), and long daily average for session duration (+22.9%; *P*=.045). Navigation flow analysis showed more traffic toward web pages related to health conditions in the redesigned version.

**Conclusions:**

Websites about stem cell research need to provide content for vulnerable global audiences. Participatory design that addresses knowledge gaps and information needs can produce better performance and engagement, which can be evaluated using Google Analytics, a common web analytics tool used by many websites. Learnings for improving the metrics regarding website identity, research updates, and clinical trials are concluded, which can inform the future design of websites seeking to engage users and provide reliable and accessible science and health information including but not limited to stem cell research and therapies.

## Introduction

### Overview

Although web-based health information is easy to access and low cost [1], it is often found to be misleading and inaccurate [2]. This is particularly true for complex information related to stem cell therapies (SCTs). Numerous studies have documented the emergence of websites that use exaggerated claims and misleading information across the globe to promote purported SCTs that are being sold to people living with a wide range of conditions—from individuals with spinal cord injury to children with autism—by commercial clinics with little to no evidence of safety or benefit [3-6]. To counter the exaggerated claims made on the web and provide better clarity for health information consumers navigating a complex information environment, international and national stem cell research organizations have developed handbooks and web-based educational resources such as the EuroStemCell resource [7,8]. In Australia, this has included placing public education and the need to meaningfully engage with health information consumers at the core of a series of major, government-funded, and stem cell science–focused research initiatives over the past 2 decades [9]. Most recently, these activities were led by the national research consortium named Stem Cells Australia [9]. Although this initiative was originally established for a 9-year period to support the clinical translation of promising basic research, it was seen as essential to continue the public education activities beyond the grant, creating an opportunity to rebuild an Australian website explicitly for public education about stem cell science.

Health information–seeking behavior is an essential coping strategy for facing illness [10], particularly for patients with chronic conditions [11]. Studies suggest that the quality, relevance, and severity of health conditions are considered in the process [12,13]. Patients’ needs are often unmatched, as the information provided is not what they want or need [14]. For SCTs, the complex landscape with commercial, biased, and inaccurate information has worsened the issue. Participatory design is an approach that has been adopted successfully in diverse medical contexts [15,16]. Many studies demonstrate that participatory design, that is, involving patients who are health information seekers in the design process, can lead to better user experiences and more accessible health information and create a great impact in relevant communities [17,18]. A core feature of public engagement strategies to support patient communities in the stem cell field has been directed at equipping patients with accurate and reliable information [8,19]. However, the form that information should take and the tools that are most effective in disseminating such information remain largely unexplored. Our approach centers participatory design as a key strategy in addressing the complex issues surrounding the design and provision of web-based information about stem cell research and therapies.

Efforts to strengthen the dissemination of trustworthy treatment information and research updates through the platform of Stem Cells Australia were and continue to be critical given the high volumes of inaccurate claims. Participatory design is recognized for delivering improved health communication outcomes [20], but few studies focus on the digital content of stem cell research and therapies. To contribute to the dissemination of stem cell information and, more broadly, other complex health information, we established the following two main goals for this study: (1) to investigate the information needs of the audience of stem cell websites to understand how a carefully crafted website can be useful for them in their journeys and (2) to empirically evaluate the outcomes of participatory design of the website after implementing the ideas emerging from the design process. These findings are helpful to understand the information needs of people considering SCTs and to generate knowledge about how to build better health information websites in this context of hype, uncertainty, and incorrect information about SCTs.

Our study on the website included 3 components. We first conducted a focus group and interviews with a purposive sample of users to determine the usefulness and drawbacks of the available web-based information about stem cells. We recruited 12 health information consumers (including patients, carers, and representatives from patient advocacy groups) who were interested in SCTs. In the second stage, drawing on their feedback, we designed and developed a new version of Stems Cells Australia, which was launched in January 2021. Finally, we collected and analyzed a total of 180 days of Google Analytics data from both the original and redesigned versions (90 days for each) to gauge the differences and improvements in the redesigned version. Google Analytics [21] is a widely used proprietary tool for recording web traffic and measuring the performance of websites. Some users may block this web analytics service using browser extensions or firewalls; however, it has been used for studying the effects of various health-related websites [22-24].

Through this empirical study, we found that websites about stem cell science and SCTs can serve as both a legitimate information source for worldwide users and a gateway to localized information. In addition, tailor-made content produced via participatory research design processes and optimizations for the website layout enables and enhances health information consumer engagement and results in better user experience. These findings are helpful to understand the information needs of health information consumers requiring information about stem cells and to generate knowledge about how to build better community-centered informational websites in general.

In the following sections of this paper, we first introduce the methods we adopted for the qualitative stage of the project and Google Analytics data analysis and then present our results. Then, we discuss the knowledge gained from this study and consider, in our conclusion, the implications of our findings for the development and design of comparable information-based health and science websites.

### Background

Stem Cells Australia was established in 2011 by the Australian Government to support stem cell researchers to develop novel diagnostic, therapeutic, and biotechnological applications [25]. The original website was designed to perform two functions: (1) to provide information about the initiative and its governance and to showcase the studies conducted by >300 scientists and students from across 18 universities and medical research organizations across the country and (2) to provide information to patients, health care professionals, policy makers, government agencies, media, and interested members of the public about developments in the field, both domestically and internationally.

Addressing misinformation about available SCTs in Australia or abroad was a core activity, given the physical, psychological, and financial risks that these unproven interventions posed for individuals and the burgeoning sector more broadly. SCT misinformation is found in different forms, including commercials targeting diverse patient groups via direct-to-consumer marketing [3,5,6], fake news with exaggerated or misinterpreted scientific results [26], criticism based on political and religious motivations [26], and unproven claims purporting the effectiveness of SCTs for new diseases such as COVID-19 [27]. Such information can lead to unrealistic patient expectations, the growth of unproven SCT markets, and biased policy discussions [28].

To respond to these issues, many of the public education resources were developed in partnership with key patient advocacy and community support groups and other international stem cell research initiatives, such as EuroStemCell and the International Society for Stem Cell Research. The original website also included an option to lodge a request for more information. More than half a million unique visits were noted by Google Analytics, with >3000 enquires received during the initial phase of its operation between 2011 and 2019.

Although the full range of the activities of Stem Cells Australia concluded at the end of 2019, there remained a clear need for a reliable and accurate source of information, given the rapid advances in this complex field and ongoing challenges around effective oversight and regulation of commercial providers and clinical translation in Australia and abroad [29]. Rather than redacting the content on the original site, funding was secured through the original consortium to develop a legacy website, designed explicitly for public education and sharing the original URL [30]. Additional funds to support the first 3 years of operation of the site were provided by the University of Melbourne, the lead university in the original consortium.

To facilitate participatory design, a research group was formed that linked expertise in IT, website design, and health information seeking with the expertise in stem cell science public education and knowledge of sociocultural drivers underpinning consumer choice about SCTs from studies conducted throughout the original initiative. Participatory design was also facilitated by the guidance provided by representatives from key Australian communities and advocacy organizations.

## Methods

**Figure 1:**
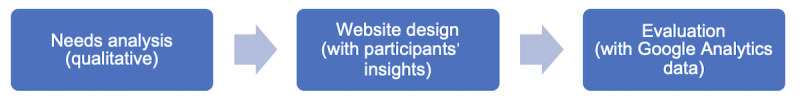
Overview of the design of this study.

This case study comprised 3 steps, namely, needs analysis, website design, and evaluation, as shown in Figure 1. This section explains our research design and the methodology used in each part of this study.

### Step 1—Needs Analysis

The purpose of our user needs analysis was to understand what stem cell information users seek and how they obtain this information. In participatory design, purposive sampling can generate maximum acceptability and usefulness, and such user sampling is used in the design of web-based interventions [31,32]. In this study, two approaches were used to recruit participants: (1) a message was sent to a purposive sample of potential participants who were on a mailing list compiled by Stem Cells Australia of members of the public who had previously contacted Stem Cells Australia for information and expressed interest in being a part of the study and (2) a call for participants was distributed via email among patient advocacy organizations and community groups and via the social media accounts of Stem Cells Australia. Participants who were aged >18 years and who had experience in using Stem Cells Australia or other websites on the internet about SCTs were included in our study.

A focus group was initially organized, and because of COVID-19 restrictions, one-on-one semistructured qualitative interviews were later conducted. The focus group and interviews lasted on average 45 minutes and were audio recorded and transcribed by a professional transcription service. Interviews were semistructured and contained questions about the motivations of users in seeking SCT information; how they searched for information; types of information sought; experiences of and views about navigating the information and resources found, including the challenges, limits, benefits, and usefulness of web-based resources; what information would be the most useful; and how that information would ideally be presented on the web. Participants received a supermarket voucher worth A$20 (US $13.22) for their time. Data were deidentified and thematically coded manually by the interviewer and cocoded by another team member using NVivo (version 12.0; QSR International) data management software [33]. A combined inductive and deductive approach was adopted to first create an initial theme list based on literature (refer to the following section), and through the manual analysis of transcripts, themes were adjusted to accommodate emergent topics and specific experiences related to people’s information needs, search preferences, and views about how SCT information should be presented on the web. The team discussed codes and dominant themes generated from the data to reach a consensus about the thematic analysis and data interpretation [34].

A total of 12 participants, including people with diversity of ages, genders, and conditions (Table 1), were involved in our study. Among these 12 participants, 6 (50%) were men and 6 (50%) were women. Regarding age groups, 8% (67/12) were aged 25 to 34 years, 25% (3/12) were aged 35 to 44 years, 42% (5/12) were aged 45 to 54 years, 8% (1/12) were aged 55 to 64 years, and 17% (2/12) were aged >65 years. Regarding education, 25% (3/12) completed high school, 25% (3/12) completed vocational education, 25% (3/12) completed undergraduate degrees, and 25% (3/12) completed postgraduate degrees. Apart from the representatives from a consumer advocacy group for stem cells, participants had at least one health condition for which SCTs could be applied. The detailed demographics of our participants can be viewed in Table 1.

**Table 1:** Demographics of participants.

ID	Gender	Age group (years)	Highest education level	Employment status	Conditions	Role
1	Male	55-64	TAFE^a^	Looking for work	SARD^b^	Family of patient
2	Female	25-34	Undergraduate degree	Casual work	Neurological disease	Family of patient
3	Male	35-44	Master’s degree	Self-employed	Cystic fibrosis	Family of patient
4	Female	45-54	TAFE	Employed part time	Chronic tick-borne disease	Patient
5	Male	65-74	TAFE	Looking for work	MS^c^	Patient
6	Female	45-54	Undergraduate degree	Employed full time	N/A^d^	Consumer advocacy group
7	Female	35-44	PhD	Self-employed	N/A	Consumer advocacy group
8	Male	45-54	Master’s degree	Employed full time	MS	Patient
9	Male	65-74	High school	Retired	Lupus	Patient
10	Female	35-44	High school	Unable to work	Breast cancer	Patient
11	Male	45-54	High school	Unable to work	Spinal injury	Patient
12	Female	45-54	Undergraduate degree	Unable to work	Spinal injury	Carer

^a^TAFE: technical and further education (a type of vocational education in Australia).

^b^SARD: systemic autoimmune rheumatic disease.

^c^MS: multiple sclerosis.

^d^N/A: not applicable.

### Step 2—Website Design

We designed the new version of the website by focusing on areas including usability, navigation, information needs, consumer journey, readability, accessibility, sense making, and knowledge development. These areas were drawn from various literature related to previous experience in building health information websites and, more broadly, the human-computer interaction discipline, which provided theoretical support for extracting web design requirements from participants’ insights. Table 2 lists these design focuses and their supporting literature.

Participant feedback related to the abovementioned design principles was examined by authors (PCP, MM, and SC). Then, the feedback was passed to a third-party web designer, who was appointed to generate web design requirements, build and host the website, and provide ongoing technical support. We anticipated that the redesigned version would produce high-quality traffic, for example, low bounce rates and more reading of information. These were the areas requiring improvements, based on the experience with the original version.

**Table 2:** Focus areas in the redesign of Stem Cells Australia.

Area	Description
Usability and navigation	Consider the text style, fonts, coloring, graphics, layout, and so on, so that they match the purposes of the website [35,36]. Consider the navigation paths within a website, which allows reaching the needed information. Use user interface elements, such as indexes, site maps, and links to establish the communication between the user interface and users’ navigation needs [37]. Understand the use of different navigation paths for accessing information and respect the preferences and the control of users [36].
Information needs	Design for different combinations of states of health information needs including demanded, undemanded, recognized, and unrecognized needs [38].
Consumer journey	Support the journeys of users with different preferences and paces, because users would like to control the order of the reading and the progression through content [36].
Readability and accessibility	Provide content that is appropriate for the context and the levels of health literacy of users [20]. Allow to find information effectively and access information efficiently, while keeping track of users’ journeys for them to return to previous information [39].
Sense making and knowledge development	Use a design that helps to understand the content and its organization (eg, providing overview and tables of contents) and allows users to broaden their knowledge (eg, support for browsing, recommended pages, and links to external web pages) [40-42].

### Step 3—Evaluation With Google Analytics

To evaluate how changes in website design influenced health information consumer behavior, we compared the Google Analytics data of both the original and the redesigned versions. Google Analytics provides a range of metrics for measuring the performance of any given website. In this study, we used several metrics provided by Google Analytics, such as the number of users, number of sessions, bounce rates, number of pages read per session, and session length (in seconds). Bounce rates measure the percentage of users who visit a website and leave without continuing to other pages on the same website in a single session [43], and this is calculated by dividing the number of users leaving without visiting other web pages by the total number of users. In this study, 2-sample *z* tests were used to validate the significance of the differences in the means of these metrics. The analysis of such data can reveal the efficacy of a website, and it is widely used in research in different disciplines [44].

In addition, to understand the patterns of topics read by users, we examined the navigation flows of the website. Navigation flows refer to the activity in which a user navigates from one web page to another (eg, clicking on a link on one page to jump to another) in any given period. Although navigation flows are informative, Google Analytics provides only their aggregated counts; therefore, statistical tests were not applied to these data. In this study, we compared the percentages of users in different website versions, because their base user counts are different. As the home page is the main starting point for users’ journeys, we focused on the navigation flows originating from the home page to other parts of the website in this analysis. Sankey diagrams were used to visualize the flows.

We compared the metrics over separate 90-day periods using 2-sample *z* tests where appropriate, straddling the transition from the original to the redesigned version of Stem Cells Australia. Data were collected between October 1, 2020, and December 31, 2020 (excluding December 9, 2020, when there was a technical issue causing no data to be recorded) for the original website and between February 1, 2021, and May 2, 2021, for the redesigned website. January 2021 was excluded because it was in the transition period, when only partial data were recorded.

### Ethics Approval

This study was approved by the human ethics research committee of the University of Melbourne (1955014.1). Informed consents were obtained from the participants.

## Results

### Insights From Participants

This section presents the findings identified from the first arm of the study (ie, the qualitative focus group and interviews), which comprised 3 main themes: needed and unwanted information, how and where to obtain information, and the information preferences of users.

#### Needed and Unwanted Information

Participants highlighted a wide range of information that they would like to see on a website about stem cell research and therapies and identified several categories they would avoid when surfing the internet. These categories of needed and unwanted information are summarized in Table 3. Users reported that they explicitly looked for the following categories of information: basic information about stem cells (such as what they are, what conditions they can cure, etc), news about research and clinical trials, information about treatments, and experiences of other people. In contrast, participants tried to avoid web-based information with a sales or marketing tone or about countries other than Australia. Participants described carefully examining the web-based information they encountered to differentiate between reliable sources and problematic content that fell in the unwanted category.

**Table 3:** Needed and unwanted stem cell information.

Types and categories		Quotations
**Needed information**		
	Basic information about stem cells	“Then my other thought was, and it just came down to basic things. What are stem cells? And what are the types of stem cells?...I always just thought stem cell was one thing. So a page on what are stem cells and what types of stem cells.” [Participant 9]
	Research and clinical trials	“I wanted to look at any studies. I wanted to find any studies of any good outcomes.” [Participant 4] “I guess I was looking for information about stem cells, how they’re applied, what clinical trials might be available...” [Participant 3]
	Treatment processes	“I guess the other way of seeing this is like this would be like to explain the process of what treatment, say the first step, what would it be, or what’s the preparation work?” [Participant 9]
	What the treatment would involve	“I would be looking for other patients’ opinions, their experience, the support that you get, what to expect from a procedure, the downtime. How it can affect your life. What you can and can’t do during the time that you’re having that sort of treatment.” [Participant 9]
	Australia-based reputable information	“And again, the trust issue was a lot of these websites were overseas and you weren't sure whether they were, for lack of a better word, scams or whether they were actually genuine.” [Participant 3]
**Unwanted information**		
	Sales or marketing pitch	“Be very careful; what they say is not necessarily going to work, And there’re a lot of scams out there.” [Participant 1] “I think like ads popped up for like, cure breast cancer naturally, but it was all a load of crap.” [Participant 10]
	False information about stem cells from foreign countries	“And again, the trust issue was a lot of these websites were overseas and you weren’t sure whether they were, for lack of a better word, scams or whether they were actually genuine.” [Participant 3]

#### How and Where to Obtain Information

Participants suggested that they used search engines (eg, Google) to find the needed information. Some sample keywords used included “Stem Cell,” “Stem Cell Therapy Australia,” and so on. In addition, internet forums were reported as another channel that health information consumers used to ask questions about SCTs and learn about other people’s experiences. Notably, despite its public popularity, our participants emphasized that they did not search for or find stem cell information on social media because it was seen as having low quality and being linked to commercial efforts to sell SCTs and therefore were considered to be untrustworthy. Table 4 lists the channels that participants used to access what they perceived to be relevant and reliable information about stem cell science and therapies and includes representative quotes.

**Table 4:** The channels for obtaining stem cell information.

Categories and channels			Quotations
**Needed information**			
	Search engine	“I just typed in ‘Stem Cell,’ I guess ‘Procedure’ And yeah just kind of went from there.” [Participant 2]	
	Forums	“I normally go into Whirlpool^a^. It’s not easy to find what they’ve been talking about, but it does help sometimes when I read what they’ve been writing on there.” [Participant 1]	
**Unwanted information**			
	Social media	“Facebook ads sent me a stem cell snake oil clinic from the Gold Coast. I absolutely went berserk.” [Focus group 1] “I would say 90% have lost about $20,000 and feel worse after six months, because they’ve gone to these inferior pop-up clinics that have targeted us on Facebook.” [Participant 4]	

^a^Whirlpool is one of the most popular internet forums in Australia.

#### Information Preference

Participants indicated that how information about stem cells was presented was also important, noting that it should be easy to find and useful. Users suggested that they used stem cell information found on the web to understand their health care options, including the possibility of curing existing conditions. They preferred information that was organized according to (1) the conditions they lived with or (2) the organs or parts of their body that were the most affected by their diseases or conditions. The organization of information in relation to the affected region or site of the body was raised in the context that not every patient knew the formal name of their conditions. It was also highlighted that the website content should be presented in plain language, which did not require expert knowledge to understand the information. In addition, the reliability and credibility of information providers were crucial, so that participants could assess and access legitimate accounts of experiences and knowledge that were reliable and trustworthy. Finally, some participants suggested the use of interactive information, such as chatbots (for users who wanted to ask questions) and videos (for people who did not want to read text), to cater to different needs of website users. Table 5 summarizes the reported preferences regarding web-based stem cell information.

**Table 5:** The preferences for obtaining stem cell information.

Preference	Quotations
Information linked to certain conditions, organs, and body parts	“Especially if English isn’t your first language, images are quite easy to relate to if English isn’t your strength. So with my mom, she doesn’t speak English very well, so it’d be easier to see, ‘okay, that’s the heart, that’s the brain’ she could you know.” [Participant 2]
Plain language	“If you read an article, and you read halfway, and you don’t understand what they say, you lose interest in continuing reading. Therefore, what you read previously is all wasted.” [Participant 10] “Again, cause if you’re new to it, sometimes the terminology can be a little bit overwhelming.” [Participant 2]
Reliable and credible information	“I was probably looking to validate, I guess, the legitimacy for stem cell as a treatment. I guess the hard part in trying to consider all the information out there is to see what has some kind of medical facts behind it...” [Participant 3] “I guess part of that you want to do is have access to someone with legitimate experience and knowledge in the area.” [Participant 11]
Interactivity	“One of the key things is how people assimilate information. Some people like to click and read. Some people like to hear it explained. My suggestion is that you actually look at multiple ways of explaining the information.” [Participant 6] “I did also watch some videos, a couple of videos, and those videos were quite good. Something I should add also, for people who are chronically unwell, sometimes it is difficult reading a lot of print and a lot of content.” [Participant 4]

### Redesign of the Website

**Figure 2:**
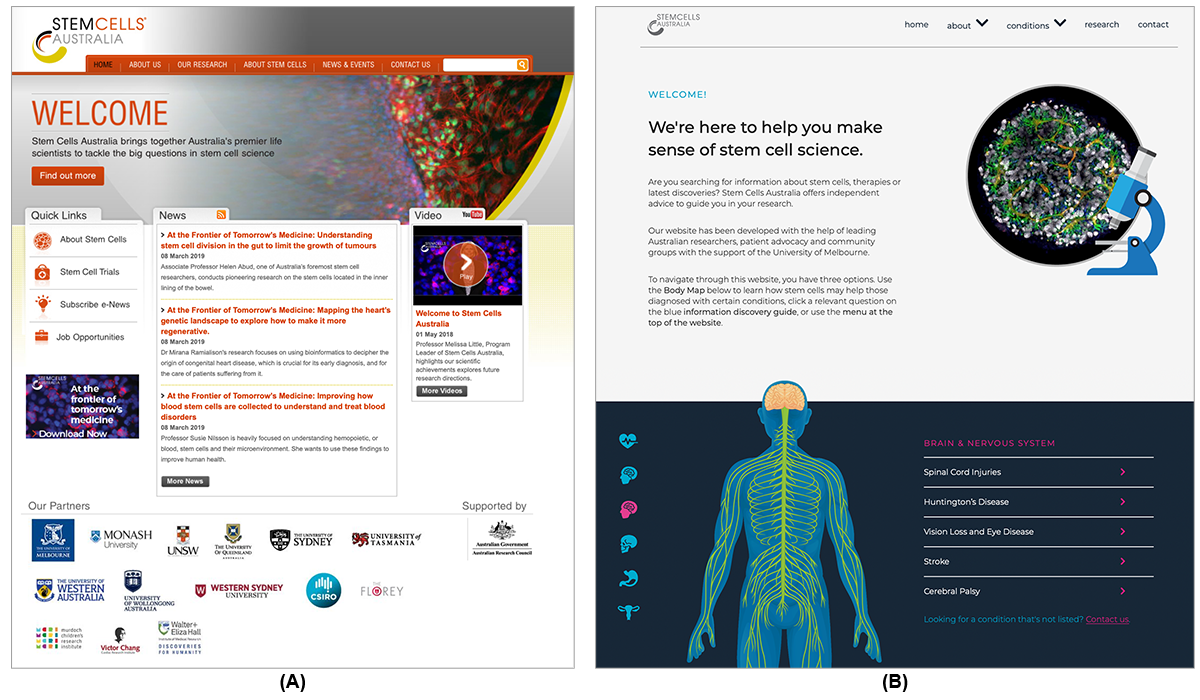
Comparison of the home pages between the (A) original and (B) redesigned versions of Stem Cells Australia. The redesigned version features condition-based navigation menus and the visualization of human systems.

Using the design principles listed in Table 2, we built a new version of the Stem Cells Australia website that aligned its content, structure, and organization with the needs and preferences of users, where feasible. Figure 2 shows the home page of the redesigned website compared with that of the original version. Users reported that they sometimes did not know the terminologies of conditions and treatments, which made finding the needed information difficult. In the new design, we used various user interface elements, including menus with condition names and visual presentation of human systems, to allow users to find and explore the information with an easy-to-understand approach.

**Figure 3:**
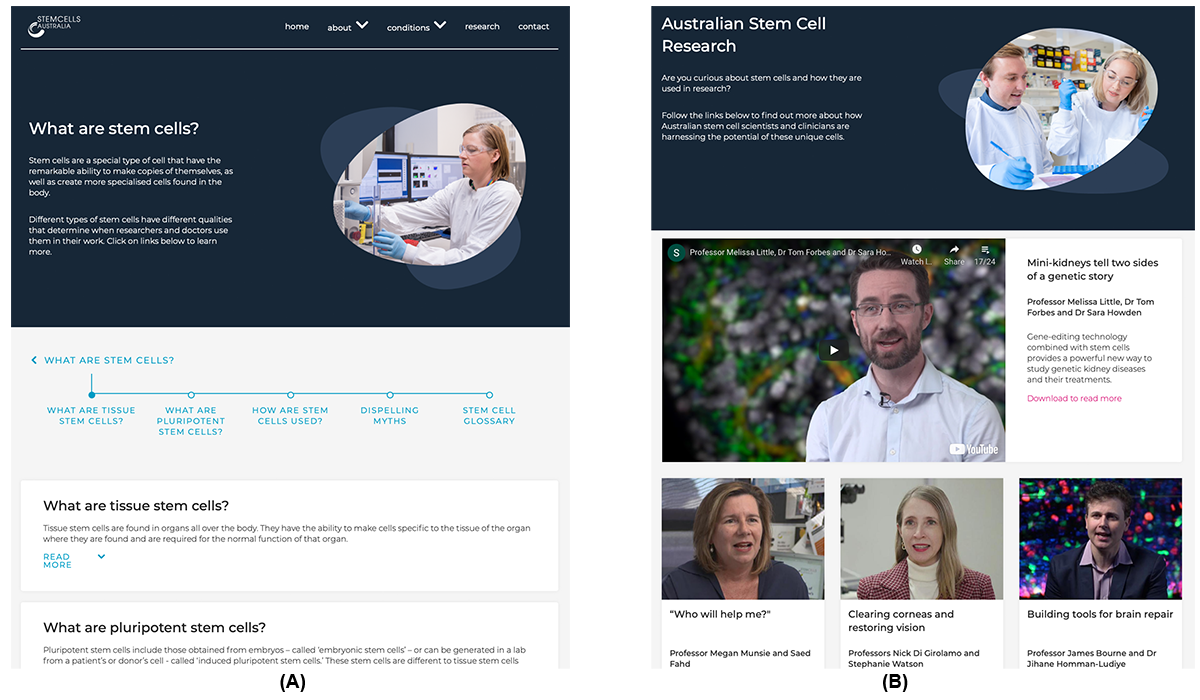
The (A) content page and (B) research section of Stem Cells Australia.

Figure 3 illustrates how we applied participants’ feedback in our design. Figure 3A displays a sample content page of the subtopics. The overall design of the site used large spacing and appropriate font sizes to improve readability and usability and to provide guidance to users during their journeys. A clear heading and a summary were provided on these web pages, to help users to understand the content before committing effort to read long, more detailed sections of text. In addition, the progress bar showed the key sections of the page, to allow users to navigate the site at their own pace and understand the current topic. Figure 3B presents the current research section of the website, where the stories of academics and researchers working in stem cell science are presented. This part of the website includes videos and articles with images aimed to present contemporary information about the latest developments in stem cell science, research, and therapies and to effectively engage patients. This was done to address the comments from participants that the original version lacked interactive content such as videos, which are widely used in modern websites, to explain in-depth knowledge.

### Evaluation With Google Analytics

In this section, we report on the results of our evaluation and the comparison of Google Analytics metrics between the original and the redesigned websites.

#### Users According to Countries or Regions

The original website recorded 7970 users, whereas the redesigned one served 3095 users during the data collection periods. Most users visiting either website version were from Australia (4801/7970, 60.24% and 1542/3095, 49.82% visiting the original and redesigned sites, respectively), with users from the United States being the next most frequent visitors. The audience for both versions also included people from multiple countries in Asia and Europe, where their primary spoken languages were not English, despite both versions providing content only in English. Table 6 shows the breakdown of the top 10 countries or regions from which the users originated in both versions.

**Table 6:** The geographic locations of users (top 10 countries or regions).

Rank	Original version			Redesigned version	
	Country or region	Users (n=7970), n (%)	Country or region		Users (n=3095), n (%)
1	Australia	4801 (60.24)	Australia		1542 (49.82)
2	The United States	609 (7.64)	The United States		368 (11.89)
3	India	341 (4.28)	China		145 (4.68)
4	The United Kingdom	160 (2)	India		108 (3.49)
5	China	109 (1.37)	Germany		50 (1.62)
6	Canada	80 (1)	The United Kingdom		48 (1.55)
7	Germany	64 (0.8)	Brazil		46 (1.49)
8	New Zealand	51 (0.64)	Canada		41 (1.32)
9	Philippines	43 (0.54)	Pakistan		34 (1.09)
10	Japan	42 (0.53)	Hong Kong		29 (0.94)
N/A^a^	Others	1670 (20.95)	Others		684 (22.1)

^a^N/A: not applicable.

#### User Metrics

As shown in Table 7, the original version had more daily users and sessions, but both these metrics declined by approximately 61% in the redesigned version (*P*<.001). The means of daily bounce rates showed a significant decrease of 97.2% in the redesigned version (*P*<.001). Regarding daily average pages read per session, the redesigned version showed a substantially high mean value of 4.09 pages per session or a 110.8% increase (*P*<.001). Regarding average session duration, the redesigned version recorded a moderate increase of 22.9% (*P*=.045). In general, the daily means of numbers of users and sessions decreased significantly with the redesigned version (*P*<.001), whereas other metrics related to user activities substantially improved at the same time.

**Table 7:** Comparison of metrics among different traffic types.

Metrics	Original version, mean (SD)	Redesigned version, mean (SD)	Change (%)	*P* value^a^
Daily number of users	87.58 (35.809)	34.01 (10.120)	−61.2	<.001^b^
Daily number of sessions	93.68 (39.026)	35.90 (11.020)	−61.7	<.001^b^
Daily bounce rate	0.72 (0.112)	0.02 (0.029)	−97.2	<.001^b^
Daily average page per session	1.94 (0.682)	4.09 (1.275)	+110.8	<.001^b^
Daily average session duration (seconds)	72.61 (46.483)	89.21 (80.777)	+22.9	.045^b^

^a^2-tailed *z* test.

^b^Significant at *P*<.05 level.

#### Navigation Flows

**Figure 4:**
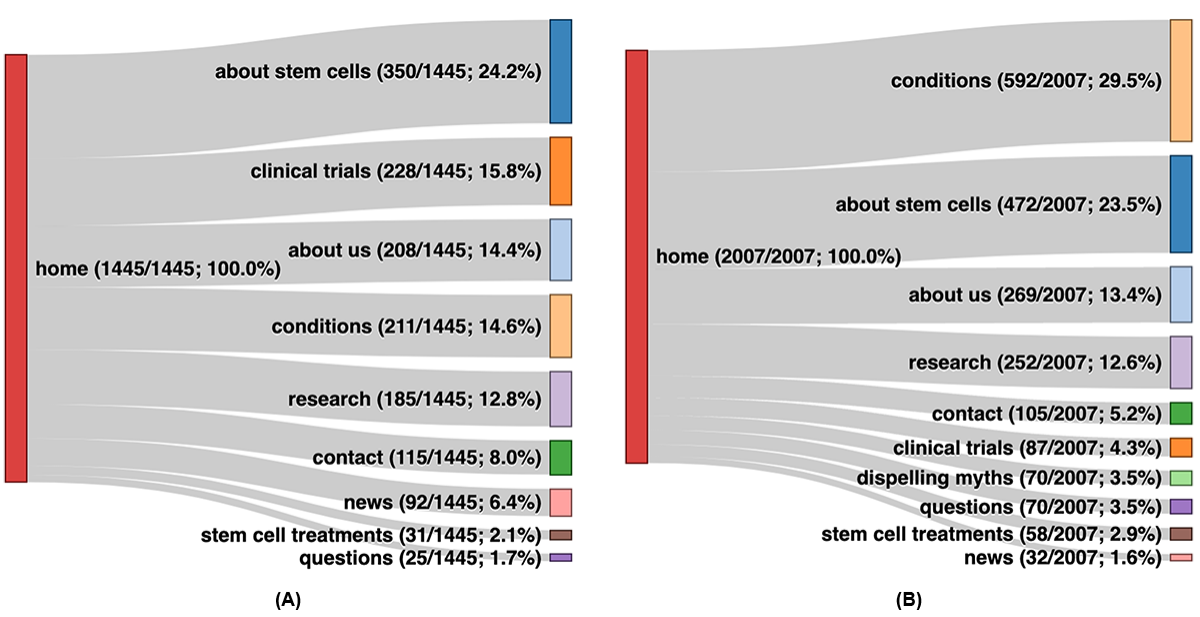
Comparison of navigation flows between the (A) original and (B) redesigned versions.

To explore the differences in user navigation behavior between website versions, we explored the navigation flows between versions. Figure 4 displays the comparison between the original and the redesigned versions during the data collection period of 90 days. First, the original version had the most traffic flowing to the *about stem cells* section (350/1445, 24.2%), whereas the section attracting the highest portion of traffic in the redesigned version was the *conditions* section (592/2007, 29.5%). The *clinical trials* web pages (228/1445, 15.8%) attracted the second highest level of traffic in the original version but only little traffic (87/2007, 4.3%) in the redesigned version. The following sections had similar ratios in both versions: *stem cells treatments* (31/1445, 2.1% in the original version vs 58/2007, 2.9% in the redesigned version), *about us* (208/1445, 14.4% vs 1269/2007, 3.4%), *research* (185/1445, 12.8% vs 252/2007, 12.6%), and *contact details* (115/1445, 8% vs 105/2007, 5.2%).

## Discussion

### Principal Findings

#### Reliable, Localized Content for Global Audiences

As identified in this study, web-based information about stem cell research and therapies is accessed by both local and global audiences contemporaneously. On the one hand, although our website originates from Australia, we have observed international visitors from many countries showing interest in our content. Using machine translation software, audiences from non–English-speaking locations can also read content written in English and obtain information about the latest developments in this field. It is a global problem that web-based information about stem cells includes unreliable, inaccurate, and non–evidence-based information via direct-to-consumer marketing on commercial websites [3-6]; therefore, it is important for legitimate websites to act as a trustworthy information source and to provide reliable information for worldwide visitors.

In contrast, participants reported that they would avoid using or relying on information from countries other than Australia and expressed that reputable local information would be helpful. In turn, the content of stem cell websites should be strengthened by including localized information to which partners such as consumer advocacy organizations, patient groups, and science communicators can contribute. This idea of customizing information based on visitors’ location is not new and is advocated across different health settings and groups, for example, for users of mental health websites [45] and for science communication during a pandemic [46]. Our study demonstrates a similar need for communicating complex messages related to stem cell science, therapies, and knowledge.

#### Positive Outcomes of Participatory Design

Following other health and medical studies [15,20], our study also illustrates the benefits of participatory design in the tailored development of web-based resources that are valuable to users who seek information about stem cells. Despite the reduced number of users, the new design has resulted in a substantial decrease in bounce rates, which suggests usability improvements. Traditionally, a high bounce rate can imply that a website has bad navigation design, irrelevant content, or low usability [47]. The low bounce rate of the redesigned version reflects that website users are able to find further information to browse and continue their journeys by navigating within the website. There is also substantial improvement in the number of pages read per session. These outcomes indicate enhanced user experience as a result of a successful participatory design process. In addition, identifying and incorporating the needs of users in the design of the site can result in strong user engagement, which is consistent with previous study about the importance of information discovery and connections among related content within a health website [18]. To further extend this concept, when most of the website content addresses the knowledge gaps of the audience, they are more likely to find something relevant through the network of links within a website and then follow the links to another web page. This can also explain the decrease in bounce rates and the high numbers of pages viewed in each session and indirectly justifies the cost and time of involving patients and users in the design process, which is an approach recommended for other website owners seeking to tailor content to user needs and preferences. Although there are many studies related to the participatory design of consumer health websites, our study was one of the few to examine the outcomes of participatory design retrospectively with the data of a live-running website.

#### Importance of Visible Identity

The branding of a health website can enable visitors to understand the identity and nature of the website, which has strong implications on the satisfaction and acceptance of users, and this can explain why users explicitly navigate to the *about us* and *contact* pages [48]. The design, tone, and branding of health websites are gaining more attention in previous health information websites for improving their acceptability [49,50], and this can be further applied to websites providing information about other health topics. Among those who explicitly seek guidance and support from health care professionals, many have reported frustration in obtaining information about whether stem cell treatments are an option for them or their loved ones [8,51]. As patients and health information consumers with complicated conditions are often vulnerable and desperate for information in a short period, an obvious identity of reliable and trustworthy owners can help them navigate the overwhelming amount of information sources and relieve the mental burden. This would also help users to distinguish valid information from misinformation on the web when the identities of the sources are clearly visible.

#### Providing Updates About Research and Clinical Trials

Studies have shown the need for disseminating research updates to patients and health information consumers [48], particularly for those living with chronic conditions, and, notably, information seekers are observed to have a high level of trust and interest in stem cell research [52]. In our study, we have seen participants reporting the importance of getting research updates and users accessing research and clinical trial information on our website. Many laypeople may not have effective channels for being up to date with the latest developments in stem cell research. This, in turn, creates opportunities for the promotion of evidence-based studies on the web while also making patients and health information consumers vulnerable to misinformation. Direct-to-consumer marketing by commercial stem cell clinics further contributes to the potential exploitation of vulnerable patient groups by selling hope to those who are desperate for options to alleviate their pain and improve their quality of life. As such, ensuring that information is constantly updated to reflect the landscape changes of SCT evidence is crucial to make such websites competitive with other websites promoting commercially driven information. The Stem Cells Australia team has connected a network of researchers to provide regular updates about their studies and secured 3 years of funding for managing the content and maintaining the technical infrastructure of the website.

#### Implications for Future Website Development

We suggest that websites that provide reliable information about stem cell research, science, and therapies can be a helpful tool to counter misinformation and misleading advertisements, particularly for people living with debilitating, degenerative, terminal, and chronic conditions and diseases, including marginalized or disadvantaged cohorts. Unsurprisingly, participants reported that they looked for accounts of the experiences of others with similar treatments via patient forums. Studies have also found that patients exploring SCTs actively seek anecdotal evidence of experimental treatments undergone by others to access information that they perceive traditional information sources to not be able to provide [53]. However, although potentially highly influential, the accuracy and applicability of such information can be limited and problematic and potentially contribute to increase in unrealistic expectations and financial loss or even endanger the lives of those people who seek non–evidence-based treatments as a result. To address people’s needs, consumer health websites can include verified stories of patients, accompanied by scientific explanations and potential outcomes. With these features, advocates can seek to share legitimate first-hand stories and experiences with communities and patient groups via the sharing functions on websites.

Others have shown preferences to look for contact details of websites and ask questions and hope to obtain tailored advice [9]. Although studies suggest that people ask questions regarding their health issues on social media [54], it is novel that patients contact website owners for enquiries. This suggests that functions such as “Contact Us” would be useful for users looking for information. In this case, websites can synthesize a section of frequently asked questions based on the questions asked by patients to enhance their content. In addition, Google Analytics can provide some insights (eg, pages with the most traffic) into what the public is seeking, and website owners can enhance the existing content and produce the missing content by observing user activities such as navigation flows.

With the rising awareness of trustworthy web-based health information, consumer-faced websites such as Stem Cells Australia should position themselves to provide neutral, accessible, nonconflicted, and scientifically accurate up-to-date information about stem cell research and what is involved to responsibly translate basic research into clinical therapy. As such, websites can incorporate the functions of research portals to proactively deliver the latest news and updates about progress in research to engage patients and potentially recruit participants for research projects. In the long term, these users could be mobilized to form communities of hope that are connected to medical research, instead of investing false hopes in unrealistic miracle cures.

### Limitations

One of the limitations of our study is that the data obtained from Google Analytics were surveyed at different periods, given the transition from the original to the revised website hosted on the same URL. We could not directly compare the performance of the 2 versions in this case, but we used consecutive periods to minimize variance. The COVID-19 pandemic and Christmas holidays may have also changed people’s behaviors including how they access health care, which may influence the use of the redesigned website. However, in Multimedia Appendix 1, we have presented additional data collected after the initial data collection, and those do not lead to substantial changes to our conclusions. Finally, our participatory design included only English-speaking participants and covered limited website features. Future studies could involve a more diverse cohort of participants (especially minority ethnic and cultural groups and non-English speakers) and explore user experience with new features arising from our design principles.

### Conclusions

This study has leveraged the opportunity to anchor the rebuilding of Stem Cells Australia using a participatory design approach, evaluated its performance, and profiled the information-seeking needs of users who are interested in accessing stem cell information. As we have shown previously, aligning complex knowledge and website structures with the information needs of the public can enhance their use of web-based information. This paper also provides an example of using Google Analytics data to evaluate health websites and profile different user groups. As Google Analytics can be easily installed and is free to use, web developers can use its data to identify the health information–seeking patterns of their audiences and, in turn, create tailor-made content. Our study has also provided several recommendations for optimizing information-based health and science websites. We hope that this paper can provide guidance to other health communication practitioners for effectively including users in participatory design processes. All these are the keys to effectively disseminating complex health knowledge, including but not limited to stem cells.

## Appendix

Multimedia Appendix 1 Additional Google Analytics data.

## Abbreviations

SCT: stem cell therapy

## Data Availability

The data collected and analyzed during this study are available from the corresponding author upon reasonable request.
