# Supplementary material for: Participatory Design and Evaluation of the “Stem Cells Australia” Website for Delivering Complex Health Knowledge: Mixed Methods Study
Source: J Med Internet Res. 2023 Jul 20;25:e44733. doi: 10.2196/44733 (PMC10401697; doi:10.2196/44733)
Supplement: Multimedia Appendix 1 [file jmir_v25i1e44733_app1.docx]

**Additional Google Analytics Data from 1 Oct 2021 to 31 May 2022**

The following table shows the Google Analytics metrics used in our study from 1 Oct 2021 to 31 May 2022, i.e., the same 7 months in the following year after we conduct our initial study.

| **Month** | **Daily Average of Users** | **Daily Bounce Rate** | **Daily Average Page Per Session** | **Daily Average Session Duration** |
| --- | --- | --- | --- | --- |
| Oct 2021 | 40.53 | 0.04 | 5.50 | 144.38 |
| Nov 2021 | 34.50 | 0.04 | 4.69 | 98.59 |
| Dec 2021 | 33.19 | 0.03 | 4.38 | 79.42 |
| Jan 2022 | 36.71 | 0.02 | 4.47 | 78.87 |
| Feb 2022 | 52.14 | 0.02 | 5.32 | 121.13 |
| Mar 2022 | 58.74 | 0.01 | 4.96 | 148.02 |
| Apr 2022 | 45.77 | 0.01 | 5.03 | 113.09 |
| May 2022 | 46.19 | 0.01 | 4.34 | 93.18 |

Based on this data, we calculate the average values and compare them with our initial data of the redesigned version of Stem Cells Australia, as shown in the table below.

|  | **Daily Average Users** | **Daily Bounce Rate** | **Daily Average Page Per Session** | **Daily Average Session Duration** |
| --- | --- | --- | --- | --- |
| Data Used in Paper | 34.01 | 0.02 | 4.09 | 89.21 |
| Average of Oct 2021 - May 2022 | 43.47 | 0.02 | 4.84 | 109.59 |

The daily bounce rate remained the same (0.02), while daily average users (34.01 -> 43.47), daily average page read per session (4.09 -> 4.84), and daily average session duration (89.21 seconds -> 109.59 seconds) were increased. This suggests that the redesigned version had better performance after the original data collection in the paper and still demonstrated better performance than the original version. This additional observation did not change our results. As such, we have validated that the initial analysis does not show obvious deviations in relation to time.
